# Specificity of serological test for detection of tuberculosis in cattle, goats, sheep and pigs under different epidemiological situations

**DOI:** 10.1186/s12917-019-1814-z

**Published:** 2019-03-01

**Authors:** J. A. Infantes-Lorenzo, I. Moreno, A. Roy, M. A. Risalde, A. Balseiro, L. de Juan, B. Romero, J. Bezos, E. Puentes, J. Åkerstedt, G. T. Tessema, C. Gortázar, L. Domínguez, M. Domínguez

**Affiliations:** 1 VISAVET Health Surveillance Centre, Universidad Complutense de Madrid, Madrid, Spain; 2 Departamento de Sanidad Animal, Facultad de Veterinaria, Universidad Complutense de Madrid, Madrid, Spain; 3 Unidad de Inmunología Microbiana, Centro Nacional de Microbiología, Instituto de Salud Carlos III, Majadahonda, Madrid, Spain; 4 CZ Veterinaria S.A., Porriño, Pontevedra, Spain; 5 SaBio (Health and Biotechnology), Instituto de Investigación en Recursos Cinegéticos IREC (CSIC-UCLM-JCCM), Ciudad Real, Spain; 6 SERIDA, Servicio Regional de Investigación y Desarrollo Agroalimentario, Centro de Biotecnología Animal, Deva-Gijón, Asturias Spain; 7 Dpto. de Anatomía y Anatomía Patológica Comparadas, Agrifood Campus of International Excellence (ceia3), Universidad de Córdoba, Córdoba, Spain; 8 Norwegian Veterinary Institute, Sentrum, Oslo Norway

**Keywords:** Animal tuberculosis, Cattle, Goat, P22, Pig, Serum antibodies, Sheep

## Abstract

**Background:**

Serum antibody detection has potential as a complementary diagnostic tool in animal tuberculosis (TB) control, particularly in multi-host systems. The objective of the present study was to assess the specificity (Sp) of an enzyme-linked immunosorbent assay (ELISA) based on the new multiprotein complex P22 for the detection of specific antibodies against the *Mycobacterium tuberculosis* complex (MTC) in the four most relevant domestic animals acting as MTC hosts: cattle, goat, sheep and pig. We used sera from an officially TB-free (OTF) country, Norway, and from a non-OTF one, Spain. The samples included sera from goats that had been vaccinated against *M. avium* subsp. *paratuberculosis* (MAP) and sheep from a herd in which *Corynebacterium pseudotuberculosis* had been isolated.

**Results:**

In cattle, the Sp ranged from 92.5 (IC95% 90.7–94) to 99.4% (IC95% 98.3–99.8) depending on the cut-off used and the origin of the samples (Spain or Norway). Sp in cattle (cut-off point 100) was significantly higher (*P* < 0.05) for Norwegian samples. By contrast, Sp in goats was consistently low at the 100 cut-off [30.9 (CI95%23.4–39.5)-78% (CI95% 68.9–85)]. A higher cut-off of 150 improved Sp in Norwegian goats [97% (CI95% 91.6–99)], but still yielded a poor Sp of 56.1% (CI95% 47.3–64.6) in Spanish goats. In Norway at the 100 cut-off the Sp was 58.3 (CI95% 42.2–72.9) and 90.6% (CI95% 81–95.6) in MAP vaccinated and non-vaccinated goats, respectively, indicating interference due to MAP vaccination. Sp in sheep was between 94.4 (CI95% 91.7–96.3) and 100% (CI95% 96.3–100) depending on the cut-off and country, and no diagnostic interference due to infection with *C. pseudotuberculosis* was recorded. Sp in pigs was 100%, regardless the cut-off point applied, and no significant differences were observed between pigs from Norway and from Spain.

**Conclusions:**

Due to its excellent Sp in pigs and acceptable Sp in cattle and sheep, this ELISA may constitute a suitable option for TB screening at herd level, particularly in OTF-countries.

## Background

Animal tuberculosis (TB) is caused by infection with members of the *Mycobacterium tuberculosis* complex (MTC), mainly *M. bovis* and *M. caprae*. This microorganism has a wide host range and involves a risk for human health [1, 2]. In cattle, TB is subjected to eradication programs but it is not only a problem for cattle; the infection has been reported in many domesticated animals and in free or captive wildlife [3, 4]. The prevalence of TB in cattle in Spain kept constant the last decade and has increased slightly in recent years [5]. The lack of recent progress of the eradication program is due to particular epidemiological characteristics [6, 7], including the role of domestic and wild reservoirs [8] and the inherent limitations of the in vivo and in vitro diagnostic techniques [9, 10]. The role of host species other than cattle, including goats, sheep and pigs, as well as badgers (*Meles meles*), wild boar (*Sus scrofa*) and deer of the subfamily cervinae, is regarded as crucial for MTC maintenance in multi-host systems [8, 11–15]. Consequently, TB control in cattle should not remain unaware of the TB status in other hosts. Therefore, the correct diagnosis of infected animals of different species becomes a need for improved TB control.

Official diagnostic tests used in cattle TB eradication schemes are based on cell mediated immunity (skin test and IFN-γ release assay, IGRA). However, there are several studies that highlight the potential of serology as a complementary diagnostic tool since new strategies and diagnostic tests are needed in order to improve the detection of infected animals, particularly in multi-host systems [9, 10]. In fact, it is possible to increase the sensitivity of the detection of positive cattle through the parallel interpretation of different tests that detect the humoral immune response (serology) and the cellular immune response [9]. In this context, serology is a fast, inexpensive alternative that allows retrospective studies and to process large numbers of samples. Several studies propose serodiagnosis as a useful screening tool for detecting infected animals in domestic species: goats [16], sheep [12], cattle [9] and pigs [17]. For this reason, new antigens and enzyme-linked immunosorbent assay (ELISA) platforms are being developed, including an in-house ELISA developed in our laboratory based on a multiprotein complex named P22 obtained by affinity chromatography from purified protein derivative of *M. bovis* (bPPD) and comprised mainly by cell surface proteins MPB70 and MPB83 [18]. This ELISA has been tested in a high prevalence cattle herd with promising results: the sensitivity was 87% (IC95% 80.3–92.5) and the combination of IGRA and P22 ELISA increased sensitivity to 98% (IC95% 92.5–99.1) [9]. In goats, the P22 ELISA showed a sensitivity of 85.3% (IC95% 76.1–91.4) before intradermal PPD injection and 100% (IC95% 97–100) when samples were obtained 15 days after the skin test [16]. However, no information about its Sp in cattle, goats or sheep has been reported, and data on Sp in pigs is limited to one study reporting 100% Sp for a sample of 88 known-negative pigs [17].

The aim of the present study was to evaluate the Sp of this ELISA based on the new multiprotein complex P22 for the detection of specific antibodies against MTC in the four most relevant domestic animal species acting as MTC hosts: cattle, goat, sheep and pig.

## Methods

### Study population

We used sera from an officially TB-free (OTF) country, Norway, and from a non-OTF one, Spain. The study was performed retrospectively with samples from cattle, goat, sheep and pig sera collected from different herds in Norway and Spain. The number, origin and characteristics of the sera used in this study are summarized in Table 1. No experimental animals were used in this study, and all handling and sampling of animals were carried out in accordance with local legislation (Royal Decree 53/2013 in Spain and The Animal Welfare Act in Norway).

**Table 1:** Number, origin and characteristics of serum samples tested with the P22 ELISA

	Norway						Spain					
	N of herds	N of sampled animals	Average of animals per herd ± SD	MAP vaccination	Last skin test (month before the study)	TB Status of the herd	N of herds	N of sampled animals	Average of animals per herd ± SD	MAP vaccination	Last skin test (month before the study)	TB Status of the herd
Cattle	212	500	66.3 ± 64.5 ^1^	NO	–	TB-free	9	1014	112.6 ± 94.7	NO	3	TB-free since 2012
Goat	50	100	144.7 ± 84.4 ^2^	YES**	–	TB-free	2	123	61.5 ± 9.2	YES	11/1*	Historically TB-free
Sheep	82	100	102.6 ± 70.0 ^3^	NO	–	TB-free	1	395	395	NO	–	No precedents
Pigs	18	100	825 ± 1221 ^4^	Not applicable	–	TB-free	2	100	50 ± 0	Not applicable	–	No precedents

* Herd 1 eleven months (historically TB-free herd) and herd 2 one month (TB-free region)

** 64 samples from non-vaccinated herds and 36 samples from herds where goats might have been vaccinated against MAP

^1^ Based on data from the Register of production subsidies as of 31 July 2015 for 201 of the herds

^2^ Based on data from the Register of production subsidies as of 31 July 2015 for 47 of the herds

^3^ Based on data from the Register of production subsidies as of 31 July 2015 for 79 of the herds

^4^ Based on data from the Register of production subsidies as of January 2014 for 14 of the herds

Norway is OTF and all samples from Norway were considered TB-free. The samples were collected as part of the surveillance programs for bovine virus diarrhea (BVD) (cattle), *Brucella melitensis* (goat), maedi (sheep) or specific viral infections (pigs) in Norway in 2015 (cattle, goats and sheep) or 2013 (pigs). Evaluation of the ELISA was carried out in 500 serum samples from cattle, 100 samples from sheep and 100 samples from pigs randomly submitted from herds. For Norwegian goats (*n* = 100), the Sp was calculated using 64 samples from counties where vaccination against *M. avium* subsp. *paratuberculosis* (MAP) was not used and 36 samples from counties where goats might have been vaccinated (Gudair; CZ Veterinaria, Porriño, Spain).

In Spain (non-OTF), the study was performed in herds without previous outbreaks of TB. All herds were considered as TB-free by the regional authorities on the basis of negative results in routine TB-diagnostic tests (skin tests and IGRA) and the absence of detection of TB-compatible lesions in abattoir inspection for more than 5 years. Cattle samples included 1014 animals. All goats (*n* = 123) had been vaccinated against MAP with a single-dose of 1 ml of Gudair. *Cornynebacterium pseudotuberculosis* was isolated sporadically in the sheep herd (*n* = 395).

### Serum sample collection

All serum samples were originally obtained by jugular (goats and sheep), caudal (cattle) or orbital sinus (pigs) venipuncture into serum separation tubes. Samples were centrifuged (1500 g for 10 min) and sera stored at − 20 °C until testing for the detection of MTC antibodies.

### *M. bovis* indirect ELISA

An in-house indirect ELISA that detects antibodies against a protein complex named P22, purified by affinity chromatography from bovine PPD [CZ Veterinaria (Porriño, Spain)] was developed. The ELISA was performed as described previously [9]. Briefly, plates were coated with P22 and then blocked with 5% skimmed milk powder solution in PBS. After three washes with PBS plus 0.05% Tween 20 (PBST), sera were added in duplicate at 1:100 dilution in skimmed milk and incubated for 60 min at 37 °C. Secondary antibody was used as reflected in Table 2 and incubated at room temperature (22–24 °C). The optimal dilution of secondary antibody was chosen based on previous titration of the antibody from 1:500 to 1:32000 in two fold dilutions and then the optimal dilution and time to develop the color were chosen for each specie. Colour was developed by adding 100 μl of o-phenylenediamine dihydrochloride substrate (FAST OPD, Sigma–Aldrich, St Louis, USA) incubated in the dark at room temperature. The reaction was stopped with 50 μl of H_2_SO_4_ (3 N) and the optical density (OD) was measured at 492 nm with an ELISA reader.

**Table 2:** Time of incubation, dilution and manufacturer of secondary antibody and developing time used in the P22 ELISA

	Secondary antibody				OPD
Species	Antiserum	Dilution	Time (min)	Manufacturer	Time (min)
Cattle	Rabbit anti bovine IGg(H/L)-HRP	1/4000	30	Biorad(Hercules, USA)	6
Goat	Rabbit anti sheep IGg(H/L)-HRP	1/2000	30	SoutherBiotech(Birmingham, USA)	15
Sheep	Rabbit anti sheep IGg(H/L)-HRP	1/2000	30	SoutherBiotech(Birmingham, USA)	6
Pig	Goat anti-pig IgG-Fc Fragment-HRP	1/20000	45	Bethyl Laboratories(Montgomery, USA)	15

Negative control sera from each species were obtained from TB-free animals previously described as MTC culture negative. These controls were included in every plate in quadruplicate. Positive controls were obtained from animals previously described as MTC-infected by culture. Sample results were expressed as an ELISA percentage (E %), calculated by the following formula: [sample E% = (mean sample OD/ 2 x mean of negative control OD) × 100]. Cut-off value was defined as the ratio of the mean sample OD to the double of mean OD of the negative control. Two cut-off points were set-up to calculate specificity (Sp): E% > 100 and E% > 150.

### *M. avium* subsp. paratuberculosis ab test (IDEXX, Maine, USA)

The *M. avium subsp. paratuberculosis* Ab Test detects antibodies against a protein extract of the mycobacteria. The ELISA was performed following the manufacturer’s instructions. Results were presented as S/P following: (sample OD - mean kit negative control) / (mean kit positive control - mean kit negative controls ODs) × 100. Samples with S/P ratios greater than or equal to 55% were considered positive.

### Statistical analysis

Statistically significant differences were evaluated using Pearson Chi-Square or Mann-Whitney U tests and 95% Wilson confidence intervals for Sp were calculated. The proportion positives observed in each herd or group (e.g. vaccinated vs. non-vaccinated) was compared by Pearson’s Chi-square test and Fisher’s exact test. The statistical analyses were carried out using SPSS Statistics 20 (IBM, New York, NY, USA) and interpreted considering a *p*-value of 0.05 to determine statistical significance.

## Results

The results of the ELISA evaluated in this study in animals from both Norway and Spain are summarized in Table 3. Inter-herd differences were only studied in Spain since Norwegian samples were randomly selected from many herds and few samples from each herd were available.

**Table 3:** Specificity and 95% Wilson’s confidence intervals of the P22 ELISA for detecting antibodies against the *Mycobacterium tuberculosis* complex at two cut-off points in different host species from Norway (A) and Spain (B)

		Cut-off 100%		Cut-off 150%	
Species	N° of animals	N^a^	Sp^b^	N^a^	Sp^b^
A)					
Cattle	500	20^*^	96 (93.9–97.4)	3	99.4 (98.3–99.8)
Goat	100	22^*^	78 (68.9–85)	3^*^	97 (91.6–99)
Sheep	100	4	96 (90.2–98.4)	0	100 (96.3–100)
Pig	100	0	100 (96.3–100)	0	100 (96.3–100)
Total	800				
B)					
Cattle	1014	76^*^	92.50 (90.7–94)	20	98 (97–98.7)
Goat	123	85^*^	30.9 (23.4–39.5)	54^*^	56.1 (47.3–64.6)
Sheep	394	22	94.4 (91.7–96.3)	7	98.23 (96.4–99.1)
Pig	100	0	100 (86.2–100)	0	100 (86.2–100)
Total	1631				

^a^Number of positive animals

^b^95% Confidence interval for specificity

^*^Significant difference (*p* < 0.05) in the proportion of negative animals of each species between Norway and Spain

### Cattle

**Fig. 1:**
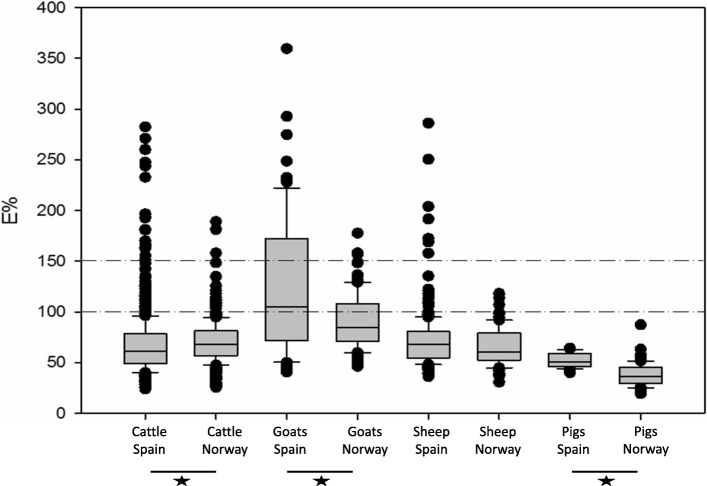
E% calculated in the P22 ELISA of different species from Norway and Spain. Boxes represent the lower, median and higher quartile ranges and outliers are represented by closed circles. The dashed lines represent the cut-off point 100 and 150%. *Significant differences observed between groups (Mann-Whitney U tests)

In cattle, the Sp ranged from 92.5 (IC95% 90.7–94) to 99.4% (IC95% 98.3–99.8) depending on the cut-off used and the origin of the samples (Spain or Norway). Specificity in cattle (cut-off point 100) was significantly (*p* = 0.0034) higher for Norwegian samples. However, when the cut-off point was set at 150 no significant differences were observed (*p* = 0.0557). The median E% value was higher in Norway (68; IQR 24.7) than in Spain (61.5; IQR 29) and significant differences were observed (M-W; U = 213,574; *p* < 0.001) (Fig. 1). Regarding cattle in Spain, the Sp varied depending on the herd. In general, Sp ranged from 95 to 99% in 8 herds. However, the Sp was lower in two herds (82 and 65%) and this reduced the overall Sp. The Sp observed in herd 8 (65.5%; IC95% 47.3–80) was significantly lower than the Sp in all other herds (1–7 and 9) (*p* < 0.001). Moreover, the Sp observed in herd 9 (82.1%; IC95%79.4–89.4) was significantly lower than herds 1–6 (*p* < 0.001). Increasing the cut-off to 150% solved the lack of Sp in these herds and no significant differences were observed. In addition, 186 animals from Spain were tested against MAP antibodies, the 76 animals positive to the TB ELISA test and 110 animals negative to the TB ELISA, randomly selected. Only one animal was positive to the MAP antibody test, belonging to the P22 negative group. Therefore, no animals with antibodies against P22 were positive to MAP.

### Goats

Specificity in goats was consistently low at the 100 cut-off (30.9–78%). The severe cut-off of 150 improved Sp in Norwegian goats (97%; CI95% 91.6–99), but still yielded a poor 56.1% (CI95% 47.3–64.6) in Spanish goats. There were significant differences in the number of positive goats between Norway and Spain regardless of the cut-off (*p* < 0.001). Regarding the median value, opposite results were obtained in goats compared to cattle. In Spain the median was higher than in Norway [105.3 (IQR 100.6) and 84.5 (IQR 36.6) respectively) and a higher dispersion was observed (M-W; U = 2666; *p* = 0.018). No significant differences were observed between goat herds in Spain. Regarding MAP vaccination of goats in Norway, the Sp was 58.3 (CI95% 42.2–72.9) in counties where vaccination might have been carried out and 90.6% (IC95% 81–95.6) in counties where vaccination was not in use, respectively (*p* = 0.002). When the alternative 150 cut-off for the ELISA was used, no significant differences were observed between groups (*p* = 0.294). In addition, the 100 goats from Norway were tested against MAP antibodies. Goats from non-vaccinated counties were negative to the test and only 7 animals out of 36 from counties where vaccination might have been carried out were positive. These seven animals were also positive to P22 ELISA.

### Sheep

Specificity in sheep was between 94.4 (IC95% 91.7–96.3) and 100% (IC95% 96.3–100) depending on the cut-off and country. The median E% in sheep was lower than in cattle and goats both in Spain (67.6; IQR 26) and in Norway (60.3; IQR 26.8) and no significant differences between countries were observed (M-W; U = 17,479; *p* = 0.081).

### Pigs

No pig tested positive either in Norway or in Spain, regardless the cut-off point applied. No significant differences were observed between pigs from Norway and from Spain. Although all pigs were negative and Sp was 100% in Norway and Spain, the median value was higher in Spain (50.9; IQR 13) than in Norway (36.3; IQR 15.70) and significant differences were observed (M-W; U = 273; *p* < 0.001). No significant differences between the two herds sampled in Spain were observed.

## Discussion

In this study we have evaluated the Sp of the P22 ELISA for the detection of MTC-specific antibodies. We found that optimal Sp (100%) was observed in pigs, whilst in cattle and sheep the ELISA had high Sp values (≥98%) using a stringent cut-off (150). Although the flock of sheep was infected with *C. pseudotuberculosis*, no evidence of interference in the serological test was observed. Contrarily, the vaccination against MAP in goats caused significant cross reactions in TB diagnosis by serology. These findings are relevant in the context of TB surveillance, especially in OTF-regions.

Previously, we assessed the sensitivity of the test in high prevalence settings of cattle and goats with promising results [9, 16]. However, no information about Sp was reported. Deciding which accuracy is acceptable and whether one prefers a higher Sp but lower sensitivity or vice versa is not straightforward. High Sp is desired in low prevalence diseases such as TB, where a false negative animal is generally preferred over a false positive one. Specificity can be modified by adjusting the cut-off value, albeit at the likely expense of sensitivity. In our particular case, the increase of the cut-off to 150% (severe cut-off) reduced the Se in cattle and goats. However, it yielded an acceptable Se of 64.5 and 75.6% in cattle and goats, respectively [9, 16]. Thus, the cut-off point must be adjusted in different epidemiological situations, and consequently vary between regions.

The P22 ELISA in cattle showed an acceptable Sp (98–99.4%) depending on the cut-off used compared with previous studies. Serological tests in cattle reported Sp ranging between 69.7 and 100% depending on the test, antigen and criteria applied [19–21]. The highest Sp was reported with the IDEXX *M. bovis* Ab test, the only official antibody test for TB approved by the OIE, yielding a Sp between 98 and 100% [20, 21].

In goats our ELISA yielded poor Sp, even in non-vaccinated herds (90.6%). There are few studies reporting sensitivity and specificity of serological test in goats. The existing serological tests for goats showed Sp values from 88 to 100% [22–24]. In this sense, the results of P22 ELISA were consistent with previous studies. It was surprising that the ELISA had low Sp in goats compared with other species. The prevalence of MAP in goats is high in Europe, including Spain [25]. In Norway, an eradication programme has been in force since 2004. Sporadic MAP cases have been reported and no MAP positive cases were detected since 2015 [26]. However, undetected infected goat herds, together with import of infected animals, may still pose a threat to Norwegian ruminants. Together with the effect of MAP vaccination, the influence of MAP infection on the test Sp should be further investigated.

Regarding sheep, little information was available in the bibliography since sheep were not considered relevant in TB epidemiology until recently [12]. Only one serological test was reported, with a Sp of 37.5% when bacteriological culture was used as gold standard and 50% when histopathology was used as gold standard [12]. This lack of Sp was explained by MAP infection. In this regard, the ELISA described here offer a higher Sp.

Finally, antibody detection tests in pigs have, in general terms, good performance and yielded similar Sp than P22 ELISA (between 98.9 and 100%) [17]. It is known that serological tests in suids are more accurate than in other species [17, 27]. The ELISA described here has a Sp similar to other tests in cattle, goats and pigs and higher Sp in sheep. However, additional studies for sheep in different epidemiological situations are needed.

Certain factors may affect the TB diagnostic performance, such as sensitization with bPPD, exposure to environmental mycobacteria, coinfection or vaccination for MAP, and infection with *C. pseudotuberculosis*. In cattle it has been proposed that injection with bPPD may affect the level of antibodies against bPPD [28]. We found significant differences between cattle from Norway and Spain. The main difference between the countries is that Spain is not an OTF country and cattle are subjected to an eradication program based basically on bPPD skin testing. The ELISA showed in cattle significant lower Sp values in Spain. This could be due to a bPPD-mediated sensitization [21, 28]. However, it could also be due to more frequent contact of Spanish cattle with environmental mycobacteria [20, 29]. Similar diagnostic interferences have been recorded regarding MAP diagnosis in goats [30, 31]. Our results support the view that vaccination against MAP strongly impacts on ELISA Sp since significant lower Sp was observed in vaccinated goats [29, 32, 33].

Some studies have suggested that *C. pseudotuberculosis* infection can interfere in TB diagnosis due to the similar antigenic compounds shared with mycobacteria included in MTC [34]. However, no significant differences between sheep from Norway and Spain were observed, despite *C. pseudotuberculosis* presence in the Spanish sheep herd. Hence, infection with *C. pseudotuberculosis* does not necessarily interfere in the diagnostic of TB by detection of antibodies in sheep. Similar results were observed in cellular mediated immunity-based tests [35].

Due to its excellent Sp in pigs and acceptable Sp in cattle and sheep, the ELISA may constitute a good option for TB screening at herd level, especially if interference by non-MTC bacteria is low or absent. In addition, serology could be used as surveillance technique for detection of specific antibodies against MTC in countries that are officially TB-free due to its high Sp and its advantages (i.e., simplicity, low cost, and requirement of minimum resources).

## Conclusions

In conclusion, the P22 ELISA, particularly in pigs, cattle or sheep, could be a cost effective, rapid and reliable tool for the screening of TB at herd level, especially in OTF countries.

## Abbreviations

bPPD: Purified protein derivative of *M. bovis*
IGRA: IFN-γ release assay
MAP: *Mycobacterium avium* complex
MTC: *Mycobacterium tuberculosis* complex
OD: Optical density
OTF: Officially TB-free
PTB: Paratuberculosis
Sp: Specificity
TB: Tuberculosis
